# A Late Diagnosis of Transthyretin Amyloidosis

**DOI:** 10.7759/cureus.21481

**Published:** 2022-01-21

**Authors:** Kristopher S Pfirman, William Newton, Collins Garst, Sharvil Patel, Jacqueline Dawson Dowe

**Affiliations:** 1 Cardiology, Geisinger Medical Center, Danville, USA; 2 Cardiology, The Medical Center - Bowling Green, Bowling Green, USA; 3 Medical Student, University of Kentucky College of Medicine, Bowling Green, USA; 4 Internal Medicine, Camden Clark, Parkersburg, USA; 5 Medical Student, Edward Via College of Osteopathic Medicine, Spartanburg, USA; 6 Cardiology, Western Kentucky Heart, Lung, and Gastroenterology, Bowling Green, USA

**Keywords:** pyp, echocardiography, end stage renal disease (esrd), diastolic heart failure, transthyretin amyloidosis

## Abstract

Cardiac amyloidosis is a rare disease caused by the accumulation of protein-based fibrils that deposit into the myocardium, causing disease. The accumulation of amyloid in the heart tissue causes the heart to become increasingly stiff, reducing compliance, with the eventual decline of the heart’s systolic function over time as the disease progresses. The restrictive physiology of the disease usually prompts investigation; however, if allowed to progress, the systolic function becomes affected in the later stages of the disease. We present a case of late-stage transthyretin-related amyloidosis (ATTR).

## Introduction

There are several different types of amyloidosis, with each derived from different proteins, including amyloid light chain (AL) amyloidosis (primary; plasma cell disorder), AA amyloidosis (wild-type amyloidosis (wt-ATTR)), and hereditary transthyretin-related amyloidosis (mATTR), seen in older individuals [1]. Although amyloidosis can affect different organs (heart, kidney, liver, spleen, nervous system, and digestive tract), the primary discussion of this case report will pertain to the deposition of amyloid in the heart. Two main forms of amyloidosis affect the heart: AL amyloidosis and transthyretin-related amyloidosis (ATTR) [1].

AL amyloidosis is caused by misfolded monoclonal immunoglobulin light chains (ALs) due to an abnormal proliferation of plasma cells in the bone marrow. Approximately, 2000 to 3000 cases of AL amyloidosis are diagnosed in the US each year [2]. In addition, two-thirds of these patients are male, and almost all of these patients are over the age of 50 [2]. In comparison, transthyretin-related (TTR) amyloidosis is associated with transthyretin, a term once known as pre-albumin. This is a protein that is primarily synthesized in the liver that has two mutated forms, a wild type and a hereditary mutated/inherited form [1].

Cardiac transthyretin amyloidosis routinely presents as restrictive physiology in the advanced stage of the disease, which only then prompts further investigation [3]. In this case report, we present a case of ATTR amyloidosis admitted with chest pain, progressive renal disease, and uncontrolled hypertension. This case is an actual example reflecting delayed identification due to confounding medical presentations and ultimately a delayed diagnosis.

## Case presentation

Our patient is a 51-year-old African American male who was presented to the hospital with uncontrolled hypertension and elevated troponins. His troponins were flat and did not trend with the typical acute coronary rise and fall pattern.

His past medical history included a remote past myocardial infarction (MI), hypertension, insulin-dependent diabetes (that was diagnosed as a likely delayed type 2, which, in retrospect, was a type 1.5 profile), peripheral vascular disease status post left lower extremity digit amputation of the hallux and second metatarsal, and chronic kidney disease (CKD) stage III. His history revealed non-adherence to his medication regimen.

He described occasional chest pains as a sharp sensation that predominantly occurred at rest. He appeared volume overloaded, including lower extremity edema and increased abdominal swelling. The patient also noted a history of myocardial infarction (MI) two years prior, where he presented similarly with elevated blood glucose and elevated blood pressure but lacked chest pain. On exam, the patient was 67.8 kg (body mass index (BMI) = 19.2), with blood sugar over 500 mg/dL, and blood pressure peaking at 261/152 mmHg. His creatinine was 1.73 mg/dL, indicative of baseline chronic kidney disease (CKD) stage III. An electrocardiogram (ECG) was performed, which demonstrated sinus rhythm with T-wave inversions in the lateral leads and anterior q waves, as well as left atrial enlargement. He was referred for an echocardiogram on account of elevated troponin and hypertensive emergency.

The left ventricle demonstrated severe concentric hypertrophy, highly suspicious for infiltrative cardiomyopathy. Unfortunately, cardiology was not consulted during this admission, the on-call cardiologist who read the echocardiogram did not suggest the possibility of an infiltrative disease process, and the patient was treated for hypertensive heart disease. The left ventricular systolic function was mildly reduced, with an ejection fraction of 50-55%. The mitral inflow and tissue Doppler flow patterns were suggestive of stage two diastolic dysfunction. There was severe left atrial dilation and moderate right atrial dilation. There was mild mitral regurgitation and trace tricuspid regurgitation. The aortic valve demonstrated mild calcification, with nodular calcification on the non-coronary cusp. There was also mild aortic stenosis, as well as trace pulmonic valvular regurgitation present. He was treated medically and sent home.

The patient’s clinical course was complicated four months after initial presentation by a foot abscess, and he was once again admitted to the hospital where he now had left foot wet gangrene due to uncontrolled diabetes mellitus and peripheral arterial disease. At this presentation, the patient was 70.31 kg (BMI = 19.2), with a blood pressure of 152/77 mmHg. High-risk femoral artery bypass for the better profusion of the left leg was planned and a Lexiscan stress test with nuclear myocardial perfusion imaging was ordered prior to surgery. The stress test revealed overall abnormal left ventricular systolic function with global hypokinesis of the left ventricle. The myocardial perfusion imaging was abnormal, with a small zone of mild intensity ischemia present in the basal to the mid-inferolateral wall.

The patient then underwent left heart catheterization and selective coronary angiography, which revealed a moderate disease of the left anterior descending, second diagonal, and first obtuse marginal arteries, as well as a chronically occluded mid-right coronary artery. After this, he finally underwent a left femoropopliteal bypass using the reverse saphenous vein approach. He was discharged to rehab but could not maintain adequate blood pressure control. 

A month later (five months after the initial presentation), he again went to the emergency department for a hypertensive crisis. At presentation, his blood pressure was 213/104 mmHg, his weight was now 78.4 kg (BMI = 22.6), labs indicated microcytic anemia (hemoglobin = 8.7 mg/dL), and creatinine was 2.3 mg/dL. An ECG was performed, which demonstrated left axis deviation, sinus rhythm, poor R-wave progression, and T-wave inversions unchanged from previous EKGs. He reported left lower leg edema and swelling, as well as mild headache and weight loss. A cardiology consult was now attained for atypical chest pain and for the management of uncontrolled hypertension.

The patient underwent another complete two-dimensional transthoracic echocardiogram. At the time of the echo, the patient was 81.2 kg (BMI = 23.0). The left ventricle demonstrated severe concentric hypertrophy. The mitral inflow and tissue Doppler flow patterns indicated stage one diastolic dysfunction. The transmitral spectral Doppler flow pattern was suggestive of impaired left ventricle (LV) relaxation. There was an abnormal longitudinal strain present with apical sparing. The left ventricular ejection fraction was normal (55-60%). The left atrium was severely dilated, and the right atrium was moderately dilated. The mitral valve leaflets appear thickened but opened well. There was trace mitral regurgitation and mild tricuspid regurgitation. There was mild aortic valve calcification and trace aortic regurgitation. There was also mild pulmonic valvular regurgitation. The inferior vena cava (IVC) was dilated with a dimension of 2.3 cm, resulting in elevated right-sided filling pressures. There was trace pericardial effusion present, with a large left pleural effusion. An atelectatic lung was noted with a jellyfish sign. Pharmacologic management was once again recommended and his anti-anginal and blood pressure medications were once again up-titrated; it was at this time during our post hoc review that the concept of amyloidosis should have been considered, unfortunately.

**Figure 1:**
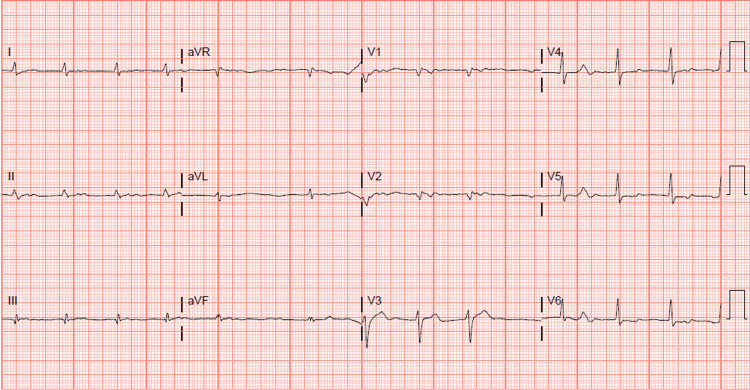
Bedside EKG

Two months later (seven months after initial presentation), the patient was admitted to the hospital with diabetic ketoacidosis and altered mental status. He had failed to follow up in the cardiology clinic during any of the time periods between discharges and readmissions. He was subsequently intubated and placed on mechanical ventilation. Cardiology was once again consulted for “abnormal heart rhythm.” After clinical stabilization and extubation, cardiology assessed the patient. A review of the telemetry demonstrated intermittent atrioventricular nodal reentrant tachycardia, paroxysmal atrial fibrillation, and episodes of junctional rhythm. The EKG obtained at the bedside, as demonstrated in Figure 1, confirmed atrial fibrillation with adequate rate control and low voltage criteria identified in the limb leads.

The patient’s clinical course was further complicated by recurrent diabetic ketoacidosis, hypertensive crisis, and gastrointestinal bleeding. After undergoing colonoscopy and esophagogastroduodenoscopy, the patient was also found to have Los Angeles grade A esophagitis, gastritis, and duodenitis, along with a 10 mm rectal polyp. The patient then underwent left adrenalectomy to remove the previously found nodule suspicious for an active adrenal adenoma given resistant hypertension.

**Figure 2:**
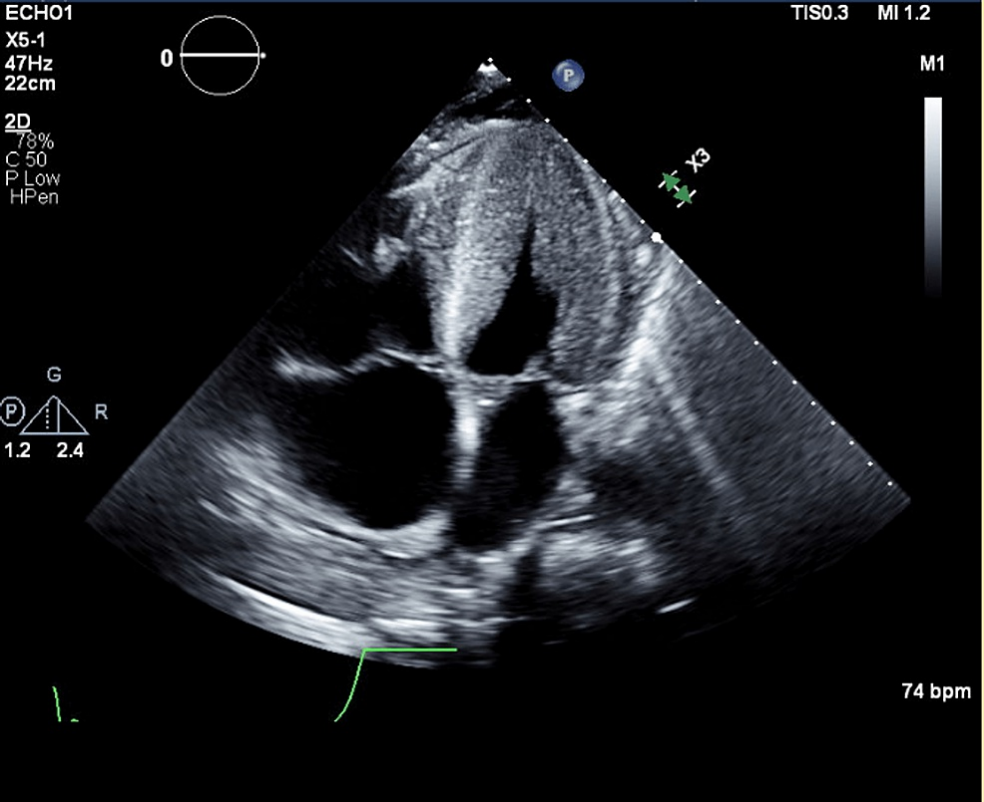
Apical 4 Chamber View - Transthoracic Echocardiogram

**Figure 3:**
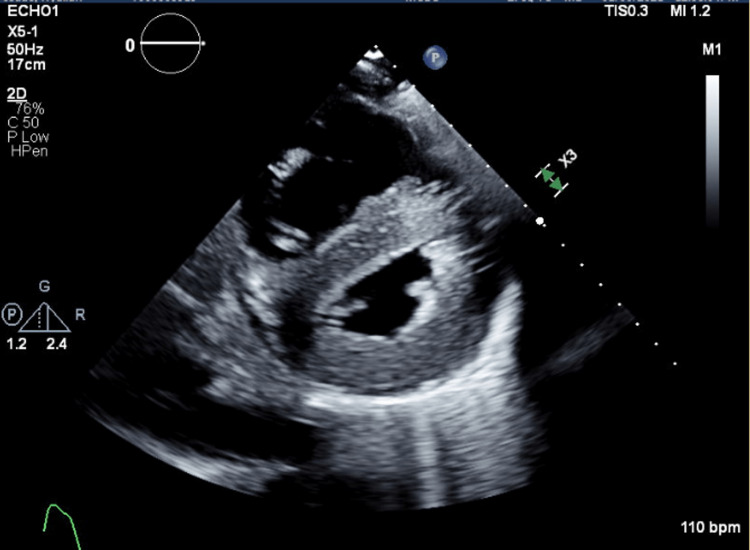
Parasternal Short Axis View - Transthoracic Echocardiogram

**Figure 4:**
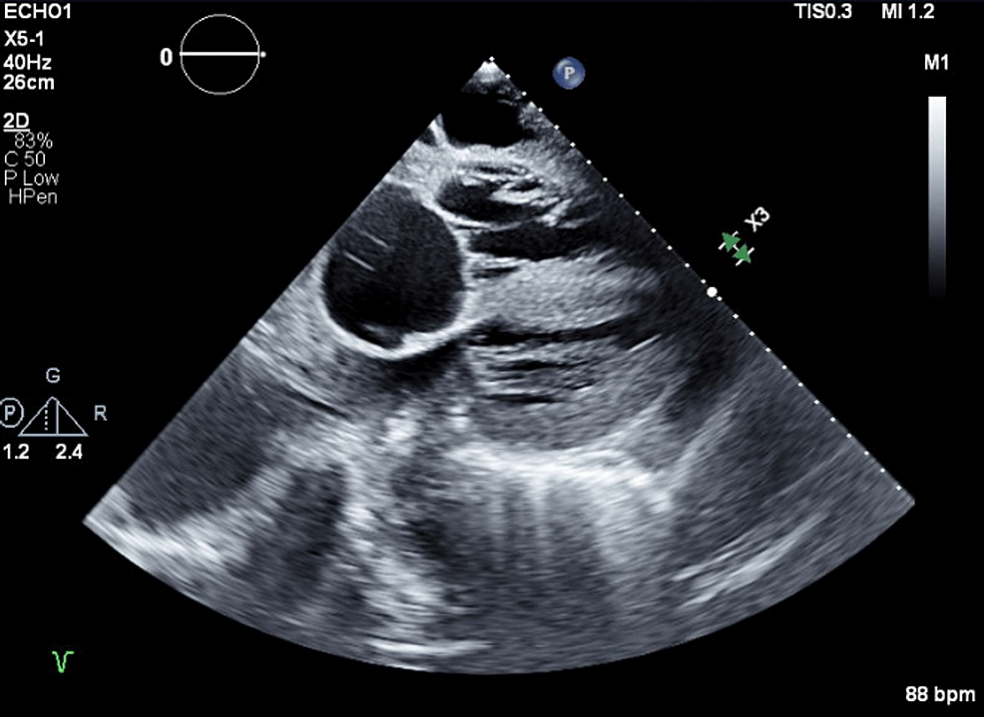
Subcostal View - Transthoracic Echocardiogram

Roughly three months later (10 months after initial presentation), as he was recovering in the long-term care unit, a complete two-dimensional transthoracic echocardiogram was ordered on account of ECG changes and persistent hypertension despite the left adrenalectomy. Left ventricular systolic function was found to be mild/moderately reduced, and the right ventricular systolic function was severely reduced. The left ventricular cavity was small, and the left ventricular wall exhibited severe concentric hypertrophy, as seen in Figure 2. The echocardiogram was compared to prior. The right ventricle was severely dilated. The transmitral spectral Doppler flow pattern was suggestive of restrictive physiology. A flattened septum was observed, which was consistent with right ventricular pressure overload, as seen in Figure 3. The right atrium was severely dilated, as seen in Figure 4. The right ventricular systolic pressure was elevated at 50-60 mmHg. There was moderate tricuspid regurgitation, and the IVC was dilated with a dimension of 2.6 cm. There was evidence of moderate pulmonary hypertension.

This is when the diagnosis of ATTR amyloidosis was first seriously entertained. This diagnosis had been overlooked in retrospect, as the patient was likely suffering from amyloidosis and not from uncontrolled hypertension due to an active adrenal adenoma. Even though multiple cardiologists had evaluated the patient throughout this time, the diagnosis of amyloidosis was protracted due to the confounding adrenal tumor and focusing on the comorbid conditions like long-standing hypertension, diabetes mellitus, and the patient’s non-adherence to medical therapy and physician appointments.

**Figure 5:**
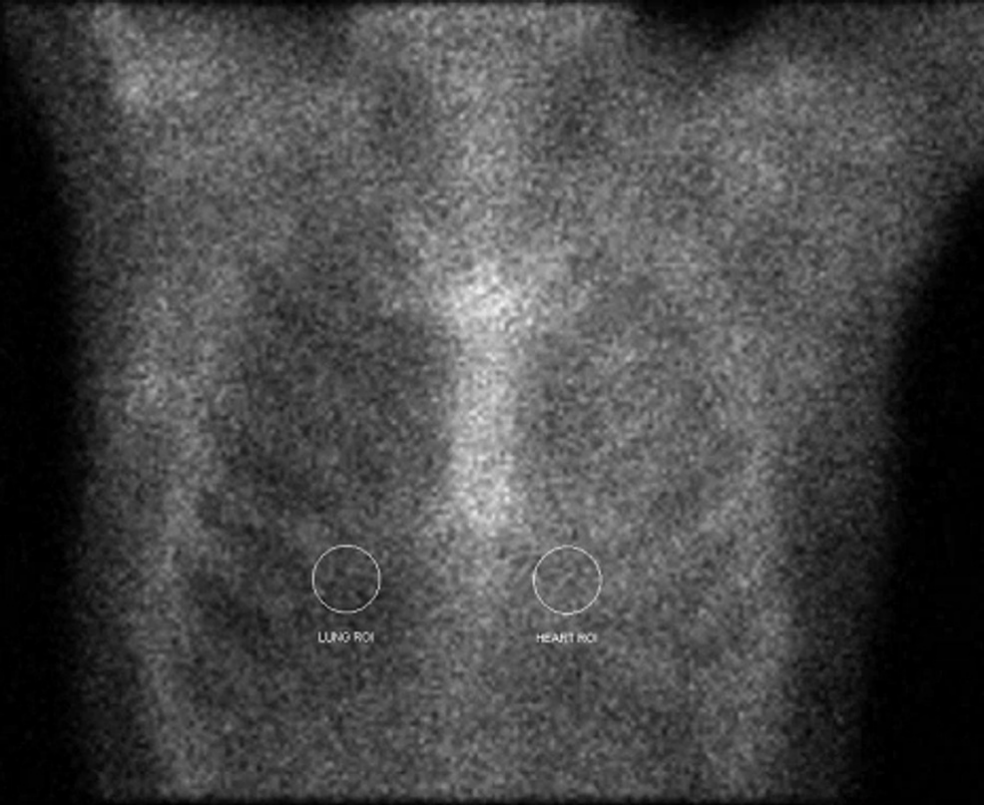
Technetium 99m pyrophosphate scan Grade 3 Myocardial PYP uptake; Heart to contralateral lung ratio of 1.65

He was then admitted to the hospital for further inpatient evaluation, where 99m Technetium-Pyrophosphate imaging was performed to evaluate for cardiac amyloidosis as seen in Figure 5. This imaging found increased activity with respect to the heart and a calculated heart-to-contralateral lung ratio of 1.65, suggestive of ATTR cardiac amyloidosis. Other complications found during his admission included recurrent pleural effusions and progressive renal failure now requiring hemodialysis. 

To further evaluate for the differential diagnosis of cardiac amyloidosis, a serum free-light-chain assay was performed. The results demonstrated high elevations of both free Kappa and Lambda light chain levels, with Kappa at 167.48 mg/dL (H) (normal = 3.3-19.4 mg/dL) and Lambda at 129.39 mg/dL H (normal = 5.7-26.3 mg/dL). The free Kappa/Lambda ratio was found to be 1.29, which falls in the normal range (normal = 0.26-1.65). These findings are confounded by the patient’s now end-stage renal disease and ongoing hemodialysis. His pleural effusions were never interrogated. Genetic testing was then to be arranged post-discharge in the outpatient setting to further assess for gene mutations associated with ATTR amyloidosis utilizing the Ambry Genetic next-generation sequencing assay. Unfortunately, the patient has yet to follow up with cardiology.

## Discussion

This infiltrative deposition of abnormal protein within the heart causes progressive and potentially fatal restrictive cardiomyopathy [3]. Protein misfolding can occur secondary to a destabilizing mutation, as seen in hereditary ATTR amyloidosis (hATTR), or due to aging, as seen in wild-type ATTR amyloidosis (wtATTR), which was formerly known as senile systemic amyloidosis (SSA) [4]. In the United States, 48% of patients with ATTR-CM are predominantly males aged 70-80 and are of African descent with wt-ATTR. Although there have been 34 discovered mutations associated with hATTR with cardiac involvement, 23% of US patients have an autosomal dominant valine-to-isoleucine substitution at 122 [4].

This results in a phenotypic expression most common in younger females of African descent [4]. This has diagnostic implications for first-degree family relatives, as they are inherited as autosomal dominant disorders [5].

Many of the early symptoms of cardiac amyloidosis are nonspecific such as weakness and fatigue, making the diagnosis often challenging and delayed. Progressive bi-ventricular wall thickening, diastolic dysfunction due to loss of cardiac compliance, and elevation in cardiac filling pressures lead to excess buildup of fluid in the body, manifesting as congestive heart failure. Associated symptoms of congestive heart failure include shortness of breath, angina, swelling of the lower extremities and abdomen, pleural effusions, and elevated jugular venous pressure [3,6].

Multisystem organ involvement can also occur. If the pericardium is involved, chronic or recurring pericardial effusions can be seen. If the coronary arteries or cardiac conduction system are involved, one may see typical angina and/or conduction abnormalities such as atrial fibrillation, complete heart block, and bundle branch blocks. The conduction disturbances can lead to clinical manifestations such as fatigue, palpitations, and syncope. Specifically, ATTR amyloid protein can infiltrate the autonomic and peripheral nervous systems. Therefore, symptoms such as paresthesia, weakness, orthostatic hypotension, diarrhea/constipation, gastroparesis, carpal tunnel syndrome, unexplained bruising, and weight loss have been known to occur. Other extra-cardiac manifestations due to infiltration and deposition would include hepatomegaly, macroglossia, and tendon rupture [3,6].

The diagnosis of cardiac amyloidosis can be based on a noninvasive approach or invasive heart biopsies. The noninvasive modalities that can suggest the diagnosis include echocardiography, cardiac magnetic resonance (CMR), EKG, and serum biomarker testing (B-type natriuretic peptide [BNP] and cardiac troponin). The finding of a wall thickness greater than 12 mm in the absence of other heart conditions, such as aortic valve disease or systemic hypertension, should warrant a strong suspicion of cardiac amyloidosis [5,7].

This is an interstitial myocardial infiltrative disease that typically manifests itself as a constellation of diagnostic features, such as concentric left ventricular hypertrophy, bi-atrial enlargement, and restrictive filling pattern on echocardiography, with characteristic findings of apical sparing on myocardial strain imaging [8]. The ECG will be discordant with the hypertrophy seen on echocardiography, being that the hypertrophic tissue is not myocardial tissue but rather an expansion of the extracellular volume with associated edema, fibrosis, protein, and collagen deposition [8].

Cardiac MRI is another diagnostic tool well-suited to evaluate for cardiac involvement of amyloidosis. CMR is more precise and reproducible in diagnosing cardiac amyloidosis than echocardiography; however, it is more expensive and less widely available. The benefit of CMR over echocardiography is the ability of CMR to identify amyloid deposits and myocardial edema with the use of ECV and native T1 times [9].

Both amyloid light chain and transthyretin amyloidosis can be assessed using cardiac MRI, but we cannot characterize or differentiate one from the other strictly using cardiac MRI alone. The amyloid protein infiltration alters the myocardial kinetics creating difficulty in nulling the myocardium. This is usually the first recognizable feature of amyloidosis during the acquisition phase. The late gadolinium sequences are enhanced with T1 recovery-focused sequences; however, because of the diffuse nature of the infiltration, there is difficulty in finding the true nulling point of the myocardium and differentiating this from the blood pool [10]. Utilization of the phase-sensitive inversion recovery times can aid in determining the true myocardial nulling time. Due to the amyloid deposition, there is an increase in both the native T1 and T2 times [11-12].

Acquiring the cardiac extracellular volume (ECV) measurement is quite useful in helping differentiate amyloidosis from other infiltrative diseases: the formula is ECV = (1 - Hematocrit) × (Δ R 1 myocardium / Δ R 1blood); where ΔR1 is (1/T1 precontrast-1/T1 postcontrast) on a 1.5-tesla magnet [13]. Out of all the infiltrative cardiomyopathies, amyloidosis demonstrates the most dramatic increase with an ECV value of 46 +/- 7% [14]. It has been noted that the more the delayed enhancement and the higher the ECV, the worse the long-term prognosis [15]. The Query Amyloid Late Enhancement score was developed to help differentiate between the two major types of amyloidosis [14]. A score of > 13 was in favor of ATTR amyloidosis [14].

Although typically, ATTR has a higher ECV in comparison to AL amyloidosis, it is worthwhile reiterating that there is considerable overlap of the ECV and elevation of native T1 times to differentiate between which type of amyloidosis is present [14]. Nevertheless, cardiac biopsy through histological identification of amyloid deposition by Congo red staining remains the gold standard for definitively diagnosing cardiac amyloidosis [3].

Genetic evaluation has opened the frontier to new treatment options as well. Until recently, only liver transplant, alone or combined with heart transplantation, was available to decrease disease progression and mortality [16].

Treatment of cardiac amyloidosis is based on a two-step approach: management of cardiac complications due to amyloid infiltration and targeted treatment to suppress new amyloid formation and deposition by inhibiting the production and stabilizing the ATTR tetramer preventing destabilization ultimately into amyloid fibrils [1]. The administration of loop diuretics and spironolactone are the primary treatment modalities for fluid status along with dietary sodium restriction for symptom relief. Beta-blockers and calcium channel blockers have little effect in treating cardiac amyloidosis [3].

In ATTR, orthotopic liver and heart transplantation was the first to have been shown to prevent disease progression and was the established treatment for patients [3,17]. The role of small-molecule drug agents has also been shown to provide therapeutic benefits, with the hopes of avoiding orthotopic transplantation.

Emerging therapies target transthyretin (TTR) deposition at various stages along the disease process. Patisiran is a small interfering RNA (siRNA) that blocks expression of both hATTR and wtATTR, has been approved for use in ATTR with polyneuropathy, and has also shown promise in ATTR-CM [16,18]. Another pharmacologic option, Tamfidis, is a selective TTR tetramer stabilizer that slows the dissociation of TTR tetramers into monomers and prevents aggregation into amyloid that has shown to lower all-cause mortality, cardiovascular-related hospitalizations, and improve quality of life in those with wtATTR-CM or hATTR-CM [19-20].

Tafamidis was the first drug to be approved for the treatment of TTR amyloid cardiomyopathy in 2019. Diflunasil, a non-steroidal anti-inflammatory drug, has also shown initial promise as a non-selective TTR tetramer stabilizer [21-22]. A small retrospective study evaluated ATTR cardiomyopathy patients being treated with twice-daily 250 mg diflunisal. Researchers found lower serum levels of TTR, cardiac troponin levels, left atrial volume index, and differences in the wtATTR global longitudinal strain [23]. The most recent systematic review in Heart Failure Reviews evaluating the use of diflunisal in ATTR cardiac amyloidosis has supported the use of diflunisal, as there was a decrease in mortality and ATTR orthotopic heart transplantation [17]. This medication has not yet achieved Food and Drug Administration (FDA) approval for the treatment of cardiac amyloidosis.

Other therapeutic targets, such as the inhibition and disruption of oligomer aggregation and the degradation and reabsorption of amyloid fibrils, have so far yielded no reassuring long-term treatment data [16].

## Conclusions

Physicians being tricked into a diagnosis other than amyloidosis is not a new phenomenon, due to the rare extracardiac manifestations and cardiac manifestations alike. The authors would encourage a “step back and evaluate” approach, as it is very overwhelming to take care of patients with such a myriad of symptoms and try to put the pieces together, especially when reviewing prior documentation, to try to prevent what happened with our patient.

This case illustrates the importance of early diagnosis of this rare, life-threatening disease, as well as the clinical course of late-stage ATTR-CM, from physicians that were not accustomed to seeing infiltrative diseases regularly. The diagnosis and treatment of ATTR-CM have advanced significantly in the last 10-20 years due to increased awareness and increased technological capabilities in genetic testing and advanced imaging as long as it is readily available. Unfortunately, most cases of cardiac amyloidosis are still diagnosed in later stages as in our patient’s case. Early intervention is the most important prognostic factor in disease progression. Unfortunately, our patient had been previously diagnosed with hypertensive heart disease and with a confounded diagnosis of hypertensive heart disease over amyloidosis in prior hospitalizations. It was only in the later echocardiograms that the question of amyloidosis was suspected. This reinforces the need to suspect alternative pathology despite the interpretation of prior physicians. This case exemplifies the classic progression of amyloidosis. The patient never followed up with his cardiology appointments and unfortunately was difficult to manage long-term. Continued work must be performed to earlier identify those at risk of developing ATTR-CM and to discover safe and effective medical treatment options. Awareness of classic symptoms is key to an early diagnosis and should direct the patient to further testing for ATTR-based therapies.
